# Social psychology: Spotting social faux pas with AI

**DOI:** 10.1038/s44271-023-00017-w

**Published:** 2023-09-18

**Authors:** Fernando Marmolejo-Ramos, Julian Tejada

**Affiliations:** 1 University of South Australia, Adelaide, SA Australia; 2 Federal University of Sergipe, São Cristóvão, Brazil

**Keywords:** Language and linguistics, Psychology

## Abstract

New research demonstrates AI, in the form of natural language models, can identify social norm violations in text and correctly distinguish the specific violated norm. This shows AI’s potential to recognize tricky social faux pas and support cross-cultural interactions.

**Figure:**
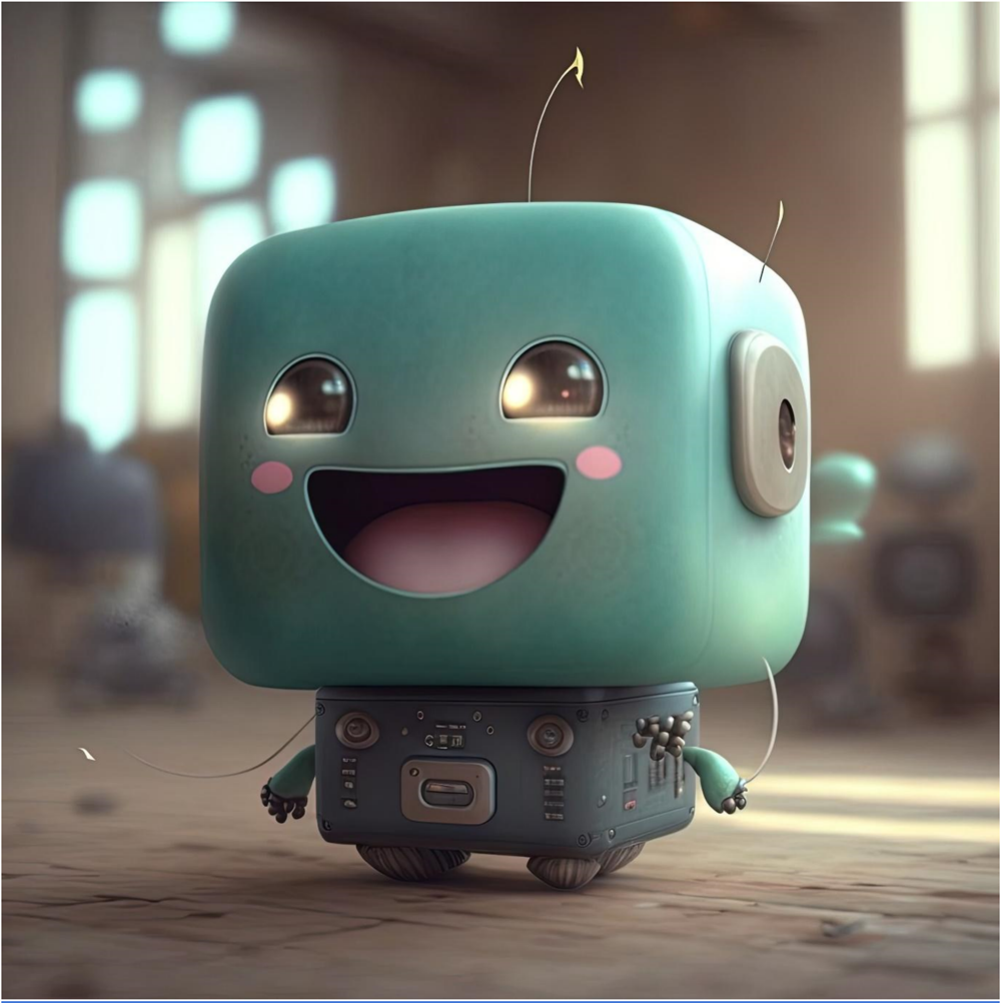
Yori Designs on Pixabay

Large language models (LLMs) are the latest artificial intelligence (AI) technology making inroads into understanding psychological phenomena. These models analyse and extract language patterns from vast amounts of text data to generate responses. One particularly interesting question is whether LLMs can appropriately recognize and handle social norms. While some social norms are universal, many are context-dependent, and it is precisely these context-dependent norms that pose the greatest difficulty in algorithmic processing, raising the question: can LLMs identify appropriate behaviours within specific social contexts.

In a new study, Yair Neuman and colleagues at Ben-Gurion University of the Negev tested whether an LLM, which was not trained to identify social norms, could classify 25 short scenarios used to measure social emotion, finding a surprising performance of 16 scenarios correctly classified^1^. They also asked the LLM to judge if a set of 8352 scenarios from a dataset of one-to-one conversations in which the speaker talks about personal feelings were examples of norm adherence or norm violation; the LLMs were better at classifying positive social emotions (e.g., pride) than negative (e.g., guilt). The researchers then identified which norm-breaking situations the model could identify and found that examples of breaking social norms of trust, caring, loyalty and conformity were best classified by the models.

This study highlights the potential of combining psychological theory with LLM to develop interpretable algorithms that can generalize social norm recognition across languages and cultures. Social cognitive scientists now have a new tool to unravel the complexities of social faux pas that transcend cultural boundaries.
